# Continuous cropping of Patchouli alters soil physiochemical properties and rhizosphere microecology revealed by metagenomic sequencing

**DOI:** 10.3389/fmicb.2024.1482904

**Published:** 2025-01-13

**Authors:** Guangtao Gu, Muhammad Zeeshan Ul Haq, Xue Sun, Jingru Zhou, Ya Liu, Jing Yu, Dongmei Yang, Huageng Yang, Yougen Wu

**Affiliations:** School of Breeding and Multiplication (Sanya Institute of Breeding and Multiplication), School of Tropical Agriculture and Forestry, Hainan University, Sanya, China

**Keywords:** *Pogostemon cablin*, continuous cropping, metagenomic, rhizosphere soil, microbial community diversity

## Abstract

Continuous cropping (CC) profoundly impacts soil ecosystems, including changes in soil factors and the structure and stability of microbial communities. These factors are interrelated and together affect soil health and plant growth. In this research, metagenomic sequencing was used to explore the effects of CC on physicochemical properties, enzyme activities, microbial community composition, and functional genes of the rhizosphere soil of patchouli. We found that this can lead to changes in various soil factors, including the continuous reduction of pH and ${\text{NH}}_{4}^{+}$-N and the unstable changes of many factors. In addition, S-PPO enzyme activity increased significantly with the cropping years, but S-NAG increased in the first 2 years and decreased in the third cropping year. Metagenomic sequencing results showed that CC significantly changed the diversity and composition of rhizosphere microbial communities. The relative abundance of Pseudomonas and Bacteroides decreased substantially from the phylum level. At the genus level, the number of microbial genera specific to the zero-year cropping (CK) and first (T1), second (T2), and third (T3) years decreased significantly, to 1798, 172, 42, and 44, respectively. The abundance of many functional genes changed, among which COG0823, a gene with the cellular process and signaling functions, significantly increased after CC. In addition, ${\text{NH}}_{4}^{+}$-N, S-CAT, S-LAP, and SOC were the main environmental factors affecting rhizosphere-dominant microbial communities at the phylum level, while pH, SOC, and AK were the key environmental factors affecting rhizosphere functional genes of *Pogostemon cablin*. In summary, this study showed the dynamic changes of soil factors and rhizosphere microorganisms during CC, providing a theoretical basis for understanding the formation mechanism and prevention of CC obstacles and contributing to the formulation of scientific soil management and fertilization strategies.

## Introduction

*Pogostemon cablin* (Patchouli), a medicinal plant in the *Lamiaceae* family, is native to Southeast Asian countries, including Vietnam, the Philippines, and Malaysia (Maheshwari et al., 1993; Swamy et al., 2010; Wu et al., 2010). Patchouli's main crop-growing regions in China are in the southern localities, particularly in Guangdong and Hainan (Wu et al., 2010). Based on its growing areas, this plant is categorized into four different types (Nanxiang, Paixiang, Zhaoxiang, and Zhanxiang) in the market of traditional Chinese medicine (Liu et al., 2023). The dried aboveground parts of patchouli are often used to treat fever, nausea during pregnancy, headaches, influenza prevention, indigestion, and their anti-inflammatory and analgesic properties (Chakrapani et al., 2013). Furthermore, patchouli volatile oil (extracted from leaves) restrains ample amounts of ketone and alcohol, crucial for the perfume industry's fragrance durability (Zhang et al., 2018).

Continuous cropping (CC) obstacles refer to continuously planting the same crop or closely related species on the same land, resulting in crop growth, yield, and quality declines (Zeeshan Ul Haq et al., 2023). This issue arises from multiple factors, including soil degradation, nutrient depletion, and the accumulation of soil-borne pathogens, all collectively weaken plant health and productivity (Zeng et al., 2020). Numerous studies have documented the adverse effects of CC on agricultural systems. For instance, research indicates that CC leads to an average yield reduction of 22%, posing a significant threat to sustainable farming practices (Wang K. et al., 2024; Wang F. et al., 2024). Additionally, continuous soybean cropping negatively impacts soil microbial communities, with increased pathogenic fungi and decreased beneficial microorganisms contributing to yield declines (Liu et al., 2022). Similarly, leguminous plants such as soybeans and peanuts, while also experiencing changes in microbial communities, benefit from the symbiotic characteristics of rhizobia, which enhance their resistance to variations in mineral nutrition, pests, and diseases to a certain extent (Jaiswal et al., 2021). Long-term CC exacerbates nutrient loss and degrades soil quality, jeopardizing agricultural productivity (Kartini et al., 2024). Studies on various crops, including *Glycine max* (Tian et al., 2020; Liu et al., 2020), *Arachis hypogaea* (Li et al., 2014), Gossypium spp. (Xi et al., 2019), and *Lpomoea batatas* (Gao et al., 2019) have shown that CC disrupts soil microbial communities, leading to significant yield reductions. Due to limited arable land and improper agricultural practices, this issue is prevalent in many farming systems in China (Lei et al., 2020). The complexity behind CCOs stems from intricate interactions within the “plant-soil-microorganism” system (Zhao J. et al., 2024; Zhao X. et al., 2024).

Modern research suggested that CC systems may lead to increased release of metabolites and leachates from plants, potentially enhancing the diversity and abundance of harmful microorganisms and promoting pathogenic bacterial proliferation (Zeng et al., 2020). This phenomenon disrupts the balance of soil microbial communities, resulting in reduced populations and diversity of beneficial and harmful bacteria, ultimately damaging the structure of the microenvironment. As these changes occur, soil microbial diversity significantly alters, impacting soil ecological functions and limiting plant growth and development (Wu et al., 2015; Tan et al., 2016). Microorganisms, as the most dynamic components of the soil ecosystem, provide crucial feedback and responses to these environmental changes (Toledo et al., 2023; Zhao J. et al., 2024; Zhao X. et al., 2024).

Changes in microbial community composition, nutrient imbalances in soil, and autotoxicity from root exudates are three primary factors associated with the occurrence of CC obstacles (Garbeva et al., 2004; Zhu et al., 2018; Liu et al., 2019). The *P. cablin* faces severe CC obstacles; however, current research primarily focuses on its molecular response mechanisms to CC, while studies on its effects on rhizosphere microbial communities, soil enzyme activities, and physicochemical properties remain relatively scarce. This research aims to (1) examine the effects of CC of patchouli on soil physiochemical properties and enzyme activities, (2) the interaction of soil properties and enzyme activities with microorganisms, and the core environmental aspects affecting the rhizosphere microbial communities of patchouli were elucidated, and (3) evaluate the variations in the diversity and structure of microbial communities in the rhizosphere under CC. Ultimately, the study seeks to recognize the impact of CC on patchouli rhizosphere soil and microorganisms, providing a theoretical basis for overcoming CC obstacles and modernizing the production of traditional Chinese medicinal materials.

## Materials and methods

### Potting material and soil samples

The experiment material for this study was the “Nanxiang” cultivar of patchouli, sourced from the germplasm source orchard of patchouli plants at Hainan University. This research was conducted at the experimental center of Ducun, Yazhou District, Sanya City, Hainan province (18° 21′23.072” N, 109° 10′7.738” E) (Supplementary Figure S1). The area is specified by a tropical marine monsoon climate, with an average annual temperature ranging from 24.9°C to 26°C. It receives an average of 2,572.8 h of sunlight annually and has precipitation of 1,100–1,300 mm/year. Healthy and pest-free branches of patchouli were taken and propagated by cuttings on a disinfected sand substrate (Yan et al., 2021). After 45 days of propagation, carefully selected patchouli seedlings with consistent growth and robust root systems. The selected seedlings were transferred to plastic pots (22c m x 20 cm x 15 cm), and each pot was filled with soil and organic fertilizer mixture in a 1:6 ratio (organic fertilizer to soil). The experimental material consisted of 120 pots containing four different cropping year soil, with each treatment having three replications and each replication containing ten pots. The pots were filled with soil (sourced from the *P. cablin* germplasm resource garden of Hainan University; 20° 3′35″ N, 110° 19′8″ E) from four different cropping years, including the zero-year (CK) cropping soil, first-year (T1) cropping soil, second-year (T2) cropping soil, and third-year (T3) CC soil. The experiment was conducted on September 3, 2023. The plants received consistent watering and management practices throughout the experiment for 100 days.

Patchouli started a key phase of persistent cropping obstacles on January 13, 2024: a rapid growth period. Four main phases define the patchouli growth period: slow growth stage, rapid growth stage, mature stage, and fully mature stage (Yan et al., 2022). Soil samples were collected from four different treatments; each treatment contains three replicates, and three pots of patchouli seedlings were randomly selected from each replicate; the rhizosphere soil (2mm from the roots) was then taken and mixed as a biological replicate sample. First, remove the plants, humus, and dry surface soil while sampling soil; then, uproot the patchouli seedlings and gently shake the roots to remove bigger dirt blocks and loosely attached soil. We divided the soil into three sections for further analysis: (1) for immediately stored the rhizosphere soil at −80°C temperature for subsequent metagenomic analysis, (2) for air-dried and sieved (2 mm mesh) soil samples for basic soil physiochemical properties, and (3) for some were stored at 4°C for enzyme activities.

### DNA extraction and metagenomic sequencing

To extract microbial DNA from patchouli rhizosphere soil, follow the manufacturer's directions employing the E.Z.N.A. ^®^ stool DNA Kit (Omega Bio-tek, Norcross, GA, USA). Metagenomic sequencing was conducted by BGI Genomics Co., Ltd., in Shenzhen, China. A Covaris M220 focused ultrasound (Woburn, MA, USA) was used to cleave genomic DNA (1 μg) for individual samples, preparing a sequencing library with a fragment length (450 bp) approximately. The BGI DNBSEQ-T7 instrument was used to sequence all samples in 150 bp paired-end (PE150) mode.

Raw sequence reads were processed using fine-tuning v0.36 (http://www.usadellab.org/cms/uploads/supplementary/Trimmomatic) for quality trimming to eliminate adapter impurities and inferior reads. Subsequently, the BWA mem algorithm (parameters: m-m-32-t16, http://bio-bwa.sourceforge.net/bwa.shtml) was operated to map quality-controlled reads to the genome of patchouli (version: hg38). Clean readings for further investigation were those devoid of low-quality data and host genome contamination.

### Quality control, assembly, and gene prediction of metagenomes

The FASTP (version 0.18.0) was used to filter the original data, resulting in clean reads for assembly analysis. The filtering standards were as follows: (1) reads having unidentified nucleotides (N) at a proportion of 10% or higher were removed; (2) reads with phred quality scores of 20 or below in at least 50% of the bases were eliminated; and (3) Reads that included adapters were obliterated. The filtered clean reads were then analyzed for assembly (Chen et al., 2018). MEGAHIT (version 1.1.1) was used to assemble the clean reads of the sample, and the continuous long sequences obtained from the assembly were called contigs (Li et al., 2015). MetaGeneMark (version 3.38) was then used to predict genes for contigs longer than 500 bp (Zhu et al., 2010).

### Gene set construction and species annotation of metagenome

Select all gene sequences with a length of ≥300 bp, using CD-HIT (version 4.8.1) to merge the sequences with a sequence similarity of ≥95% and read coverage of >90% into a cluster, and select the best sequence in each cluster (Fu et al., 2012). Long sequences are used as representative sequences (Unigene). Use Bowtie (version 2.2.5) to re-align the reads to Unigene and count the number of reads (Langmead and Salzberg, 2012). Filter the genes whose read support number is <2. The final Unigene obtained is called a non-redundant gene set. The Unigene sequence was compared to the Nr library, and the species annotation of the gene was obtained based on the MEGAN software (version 6.19.9) (Huson et al., 2011) LCA (Lowest Common Ancestor) algorithm (Huson et al., 2007); based on the gene abundance, the species abundance was obtained.

### Soil physiochemical properties and enzyme activities

A soil and water deferment (1:2.5, weight/volume) was made to assess the pH of the soil with a pH meter instrument (Model: SevenDirect SD20, Mettler Toledo, Zurich, Switzerland). Soil organic carbon (SOC) was determined using the potassium dichromate volumetric and external heating methods. Available potassium (AK), available phosphorus (AP), ammonium-nitrogen (${\text{NH}}_{4}^{+}$-N), and nitrate-nitrogen (NO_3_-N) were determined using acetic acid ammonia extraction and flame photometry (Paul et al., 2024), the molybdenum antimony colorimetric method (Li J. et al., 2023; Li L. et al., 2023; Li Z. et al., 2023), indophenol blue colorimetric method and a flow analyzer, and hydrazine sulfate reduction method, colorimetric, and flow analyzer (Mo et al., 2021), respectively. Soil enzyme activities were measured following the protocol with the respective assay kits (Suzhou Geruisi Biotechnology Co., Ltd. China). For soil catalase (S-CAT) and polyphenol oxidase (S-PPO), 0.10 g of soil was used, while 0.05 g was used for soil ß-glucosidase (S-ß-GC), N-acetyl-β-D-glucosaminidase (S-NAG), and leucine aminopeptidase (S-LAP). Samples were centrifuged under specific conditions: S-CAT, S-ß-GC, and S-NAG at 9,711×g for 10 min at 25°C; S-PPO at 9,711×g for 5 min at 4°C; and S-NAG additionally at 4,316×g for 5 min at 25°C. The supernatant was subsequently collected for enzymatic activity determination and absorbance readings were taken at specific wavelengths: S-CAT (Cat. No. G0303F) at 510 nm, S-PPO (Cat. No. G0311F) at 475 nm, and S-ß-GC (Cat. No. G0312F), S-NAG (Cat. No. G0321F), and S-LAP (Cat. No. G0329F) at 405 nm.

### Statistical analysis

Statistical software was applied to construct an Analysis of variance (ANOVA) based on the Waller-Duncan and Tukey tests to study the significance of alterations in soil properties and enzyme activities at different locations. GraphPad Prism (version 8.0.1) visualized the comprehensive microbial distribution. The stack diagram was created using Wekemo Bioincloud (https://www.bioincloud.tech). Other analyses were conducted using R (version 4.4.1). NMDS (non-metric multidimensional scaling) analysis based on Bray-Curtis distance and hierarchical clustering were employed to determine the variations in composition (microbial community) in CC of patchouli, using the “Vegan” package. The “pheatmap” program was employed to conduct the analysis, resulting in a heat map depicting the functional gene abundance and dominant microorganisms at the genus level in the sample microbial community. The “ggplots” package was employed to generate boxplots of the most prevalent microorganisms at the phylum level. The “igraph” package generated a phylum-level correlation network diagram of dominant microorganisms and COG genes. The “linkET” was utilized to conduct a mental-test analysis of the phylum-level dominant microorganisms and environmental factors. Referring to Lai Jiangshan's method, the impression of environmental aspects on microbial communities and functional genes was explored through redundancy analysis (Lai et al., 2022). To reduce the collinearity of independent variables, variables with the most significant VIF (variance inflation factor) were gradually eliminated until the VIF of all remaining variables was <20. Hierarchical partitioning was then performed to quantify the explanatory degree of different independent variables. Similarly, the “vegan” package drew RDA diagrams of microorganisms and functional genes at the phylum level. The VPA (variance partitioning analysis) was steered to determine the role of soil properties and enzyme activities in the diversity of dominant microbial communities, and “rdacca.hp” was used for variance decomposition. In addition, the “Hmisc” program was used to create a co-occurrence network established on the rank coefficient (r) of patchouli rhizosphere microorganisms at the phylum level. Gephi version 0.9.2 was used to visualize the network, with each node and edge representing a specific species and a substantial association between the two species. The degree of the distinctive topological characteristics indicated the number of edges that connected each node to the remainder of the network. Lastly, network stability was assessed by randomly deleting and repeating nodes to see how rapidly robustness declined. The natural connectedness of the nodes measured network resilience in a static network.

## Results

### Soil physiochemical properties and enzyme activities

The different CC years altered patchouli plant rhizosphere soil's properties and enzyme activities (Table 1). These obstacles significantly altered the soil pH and ammonium nitrogen (${\text{NH}}_{4}^{+}$-N), which decreased over the cropping years. In CC, soil pH decreased by 1.5%, 2.7%, and 3.9% in the first, second, and third years of CC, respectively, compared with CK. Meanwhile, soil polyphenol oxidase (S-PPO) and available phosphorus (AP) increased over the CC years. Moreover, soil organic carbon (S0C), available potassium (AK), soil catalase (S-CAT), soil-β-Glucosidase (S-β-GC), soil-Leucine aminopeptidase (S-LAP), and soil N-acetyl-β-D-glucosaminidase (S-NAG) showed a significant increase in first 2 years of CC. It decreased in the third year of cropping, while nitrate nitrogen (NO_3_-N) has vice versa.

**Table 1:** Patchouli rhizosphere soil properties in the different CC years.

**Soil properties**	**CK**	**T1**	**T2**	**T3**
pH	8.18 ± 0.03a	8.05 ± 0.06b	7.96 ± 0.01bc	7.86 ± 0.01c
SOC (g/kg)	12.82 ± 0.49d	40.08 ± 0.81a	38.06 ± 1.27b	35.95 ± 1.29c
${\text{NH}}_{4}^{+}$-N (mg/kg)	12.64 ± 1.11a	12.62 ± 0.90a	11.49 ± 0.94a	10.35 ± 1.33a
NO_3_-N (mg/kg)	9.71 ± 0.42b	17.16 ± 0.98a	9. ± 0.43b	18.79 ± 0.62a
AP (mg/kg)	52.85 ± 0.50d	137.67 ± 0.52c	147.38 ± 0.77b	167.79 ± 0.52a
AK (mg/kg)	139.38 ± 1.46d	184.09 ± 0.70c	629.75 ± 1.37a	530.38 ± 0.45b
S-LAP (nmol/h/g)	150 ± 1.86d	654 ± 2.63c	1,035 ± 3.56a	732 ± 2.87b
S-NAG (nmol/h/g)	39 ± 1.77d	141 ± 3.47b	279 ± 3.52a	127 ± 1.80c
S-β-GC (nmol/h/g)	68 ± 1.58d	192 ± 1.72c	405 ± 3.61a	282 ± 3.02b
S-PPO (nmol/h/g)	142 ± 1.80d	279 ± 1.76c	324 ± 4.02b	464 ± 3.85a
S-CAT (μmol/h/g)	49 ± 1.37d	353 ± 3.32b	434 ± 3.08a	327 ± 1.48c

Patchouli plant continuous cropping soil represents CK (zero-year cropping soil), T1 (one-year cropping soil), T2 (two-year cropping soil), and T3 (three-year cropping soil), respectively. The pH for soil pH, SOC for soil organic carbon, ${\text{NH}}_{4}^{+}$-N for ammonium nitrogen, NO_3_-N for nitrate nitrogen, AP for available phosphorus, and AK for available potassium, respectively. Similarly, soil enzyme activities, S-LAP for soil-leucine aminopeptidase, S-NAG for soil N-acetyl-β-D-glucosaminidase, S-β-GC for soil-β-Glucosidase, S-PPO for soil Polyphenoloxidase, and S-CAT for soil catalase, respectively. Data is represented by mean values ± standard deviation. The various lowercase characters in the table represent significant differences (p < 0.05).

### Soil microbial abundance and composition

**Figure 1:**
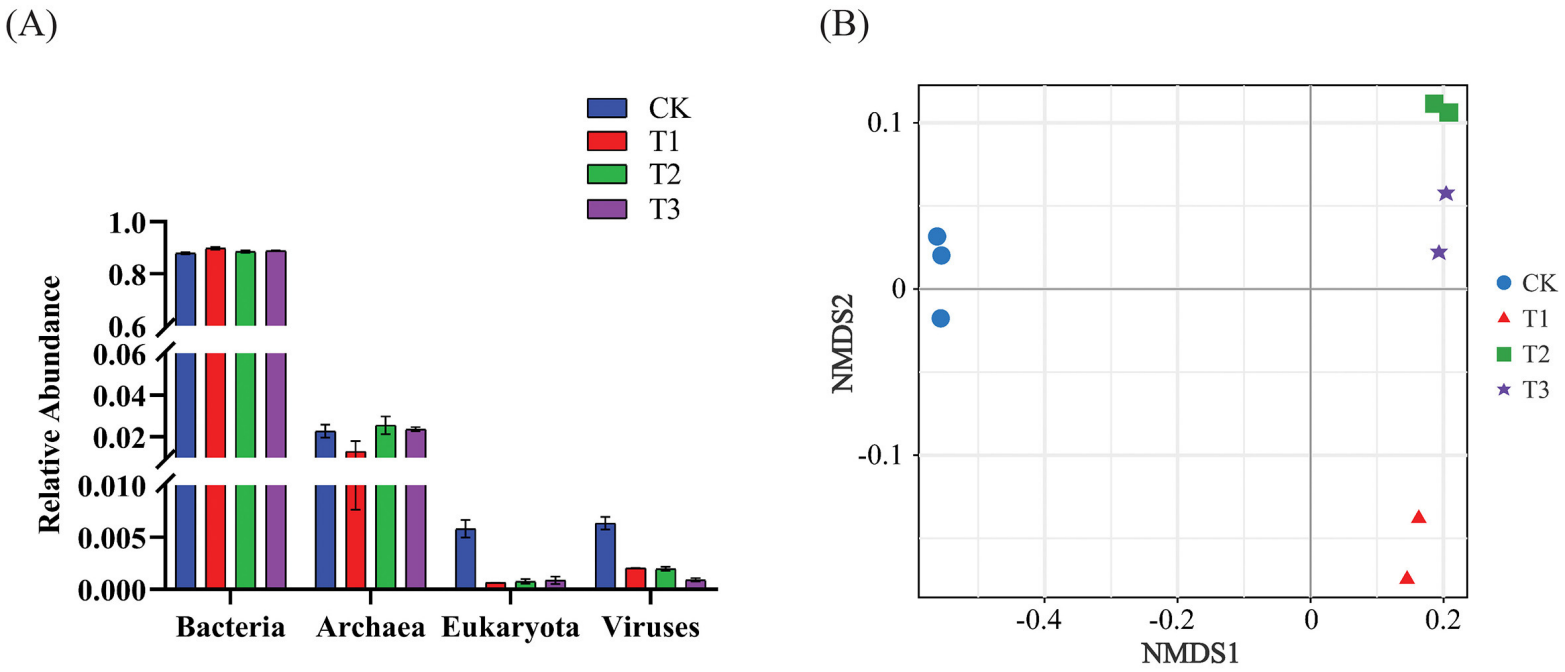
CC impact on soil microbial community composition and classification. **(A)** The classification of bacteria, archaea, eukaryotes, and viruses at different CC years was analyzed (*n* = 4). **(B)** NMDS (non-metric multidimensional scaling) analysis based on Bray-Curtis differences showed significant species-level differences in soil microbial composition. The stress value indicates the model's applicability. The model is deemed a good fit (*P* < 0.001) when the stress value is <0.05.

**Figure 2:**
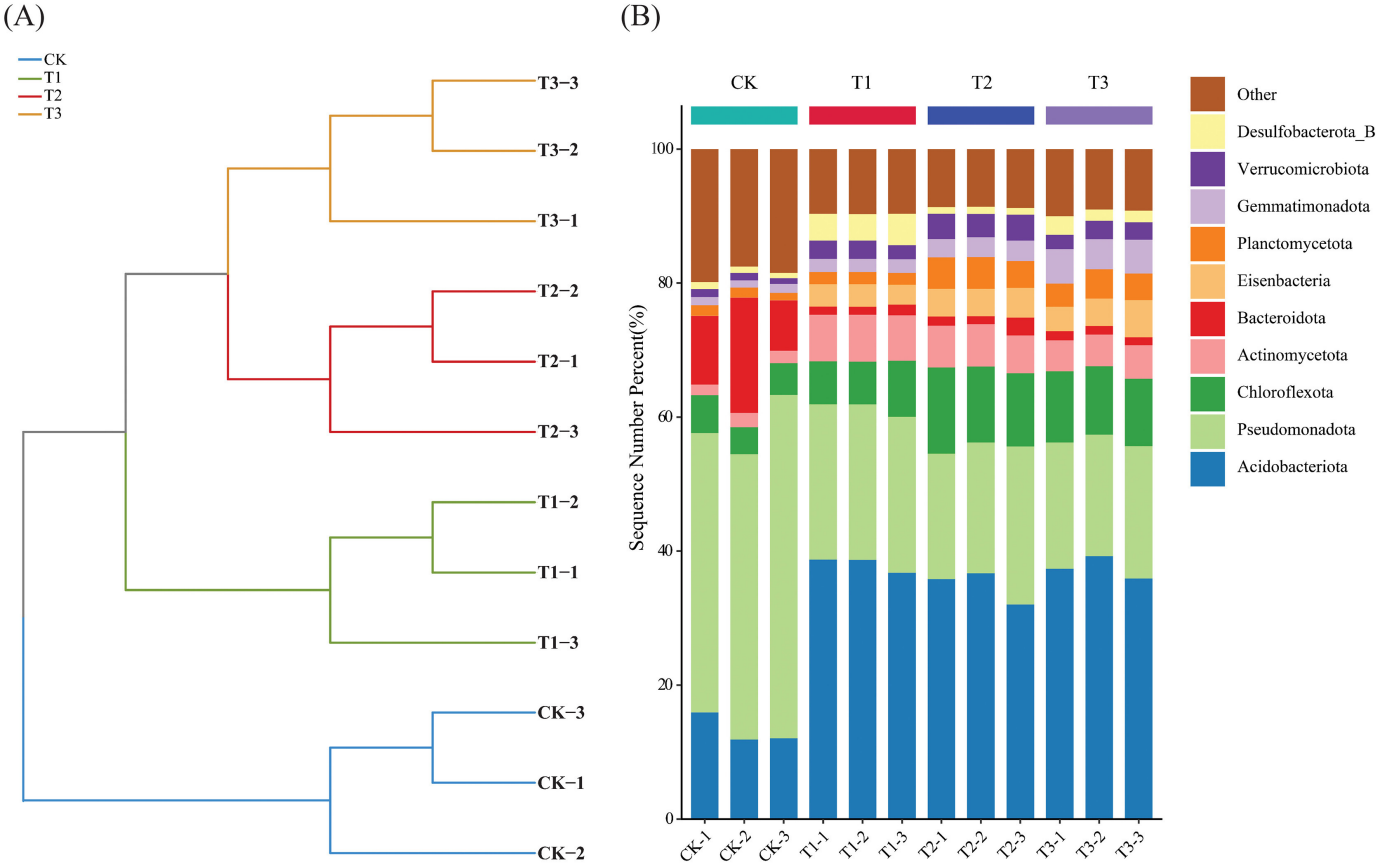
Hierarchical clustering analysis of soil microbial communities based on Bray-Curtis distance **(A)**, stacked plot of soil microbial abundance based on phylum level **(B)**.

**Figure 3:**
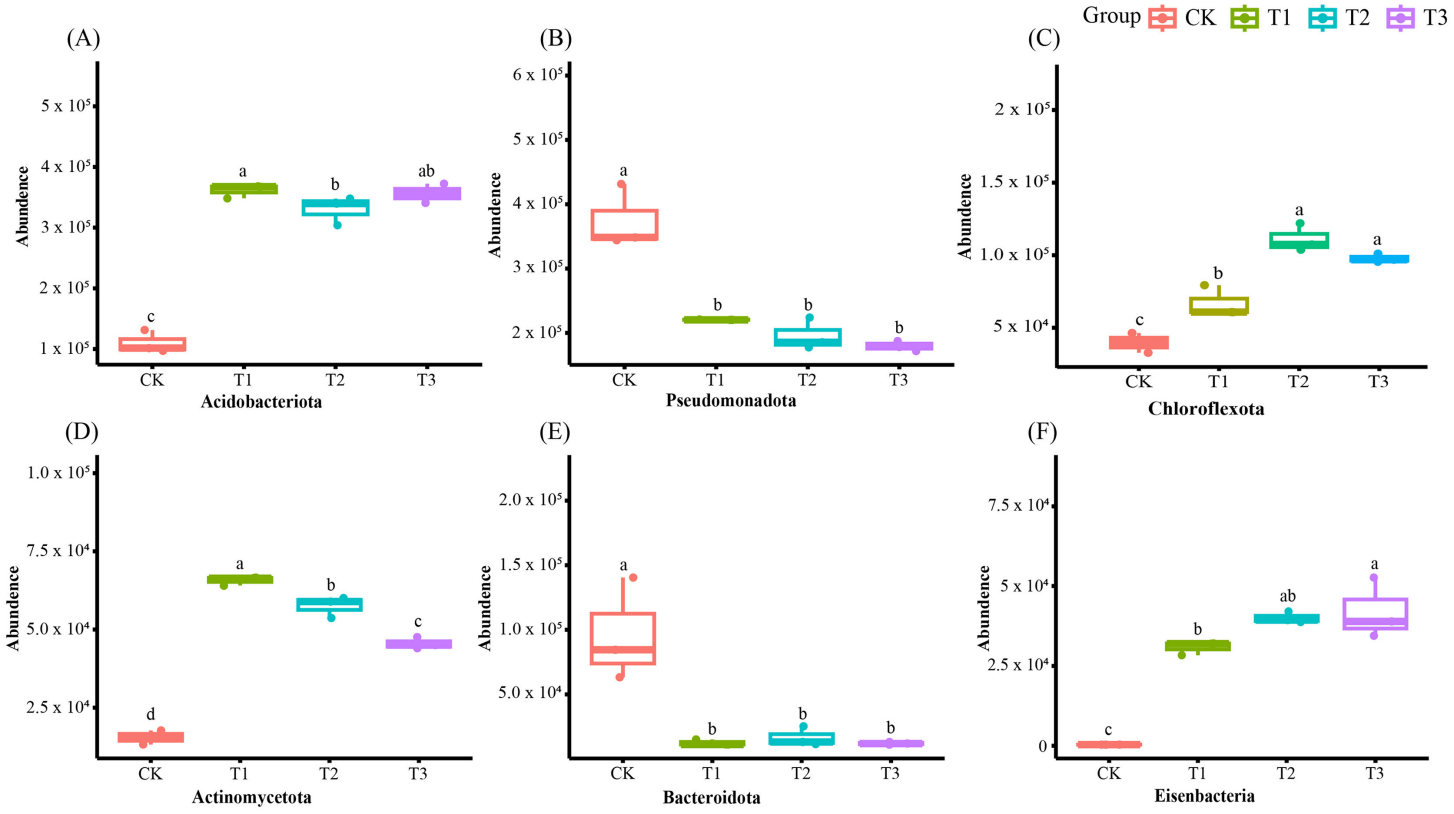
Soil microbial communities abundance at phylum level changes in CC of patchouli. Due to the long-term CC, the abundance of Acidobacteriota **(A)**, Pseudomonadota **(B)**, Chloroflexota **(C)**, Actinomycetota **(D)**, Bacteroidota **(E)**, and Eisenbacteria **(F)** has changed. Different alphabets showed notable variations (*p* < 0.05, *n* = 4).

**Figure 4:**
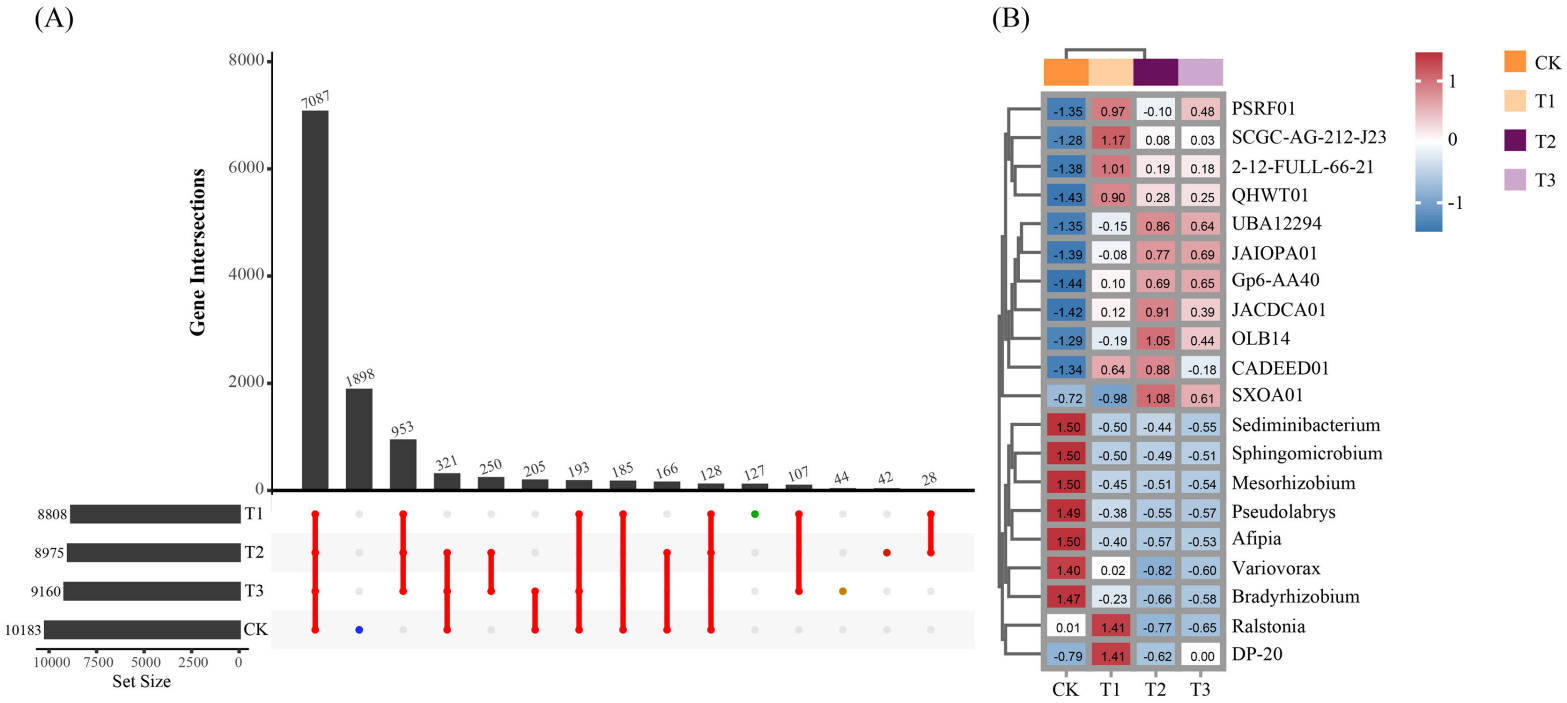
Soil microbial community domain genera affected by CC IN patchouli plant. **(A)** The Upset graph shows common and unique microbial community characteristics at the genus level. **(B)** The heat-map shows the differences between the Top 20 dominant microbial community at the genus level.

After removing human and host genome sequences, the total soil microbial community of the 12 samples accounted for 91.38%, including bacterial communities were dominant (RAavg; average relative abundance, 88.8%), followed by Archaea (2.11%), viruses (0.27%), and eukaryotes (0.19%) (Figure 1A). Whereas among the four treatments, there was no significant difference in the relative abundance of the bacterial communities. The RA of Archaea was highest at T2 and lowest at T1. Meanwhile, the number of viruses gradually decreased with the years of CC. Moreover, the RA of eukaryotes was highest in CK, decreased in T1, and then increased slowly with CC years. In addition, the microbial structure of the four plots differed significantly at the species level (Figure 1B). Among the four different soil cropping years microbial communities, the main microorganisms at the phylum level are Acidobacteriota, Pseudomonadota, Chloroflexota, Actinomycetota, Bacteroidota, and Eisenbacteria (Figure 2B). Hierarchical clustering analysis of the RA of soil microbial communities based on Bray-Curtis distance showed significant differences in microbial abundance between CK and CC soils (Figure 2A). Compared with CK, the RA of Acidobacteriota, Chloroflexota, Actinomycetota, and Eisenbacteria in CC soil increased, while Pseudomonadota and Bacteroidota's RA decreased. In addition, the RA of other microorganisms in soils planted with patchouli continuously showed significant differences at the phylum level compared with CK. CC significantly increased the abundance of Acidobacterota (Figure 3A), demonstrating that the rhizosphere soil structure contributed to enhanced resilience. The abundance of Pseudomonadota gradually decreased with CC, reaching the lowest value at T3 (Figure 3B). The abundance of Chloroflexota gradually increased but decreased significantly at T3 (Figure 3C). The abundance of Actinomycetota increased substantially at T1 but progressively decreased with CC (Figure 3D). Similarly, the abundance of Bacteroidota was highest during CK and significantly reduced after CC (Figure 3E). The abundance of Eisenbacteria was lowest at CK but gradually increased with CC (Figure 3F). Metagenomic analysis identified 10,183, 8,808, 8,975, and 9,160 microbial genera in CK, T1, T2, and T3, respectively. Among them, 1898, 127, 42, and 44 microbial genera were unique to CK, T1, T2, and T3, respectively (Figure 4A). The heat map analysis provided a more detailed examination of the variations in the prevalence of microbial species belonging to the top 20 dominating genera in the presence of RA under CC circumstances (Figure 4B). Among them, the RA of Sediminibacterium, Sphingomicrobium, Mesorhizobium, Pseudolabrys, Afipia, Variovorax, and Bradyrhizobium in CK was significantly higher than in CC soils. On the contrary, PSRFO1, SCGC-AG-212-J23, 2-12-FULL-66-21, QHWTO1, UBA12294, JAIOPA01, Gp6-AA40, JACDCA01, OLB14, and CADEEDO1 have higher abundance in CC soil than CK.

### Network analysis of high-abundance microorganisms and functional genes

**Figure 5:**
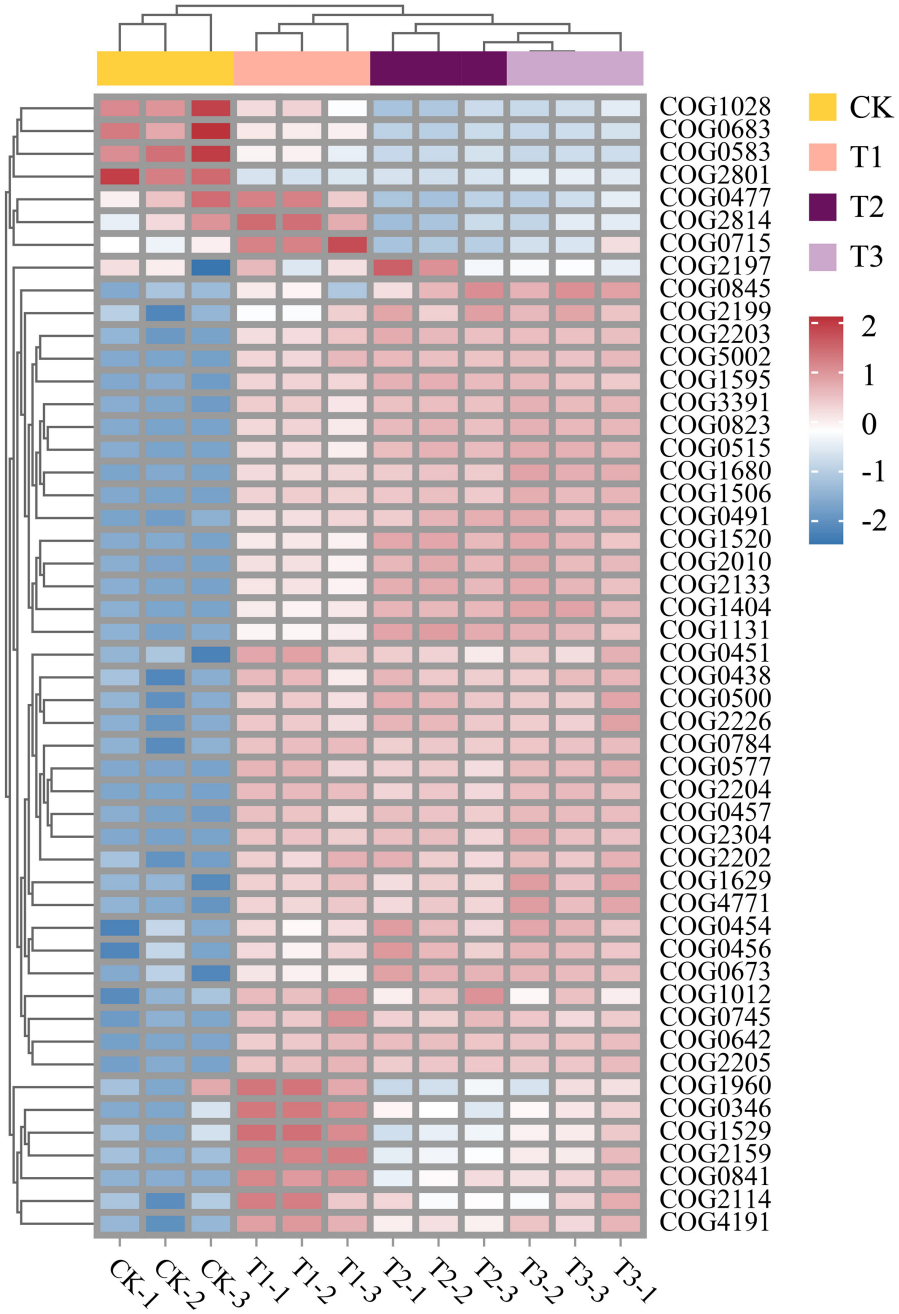
Heat map of functional gene abundance (eggNOG, top30) in microbial communities at 4 soil sampling points. COG ID, function, description, and categories are shown in Supplementary Table S1.

**Figure 6:**
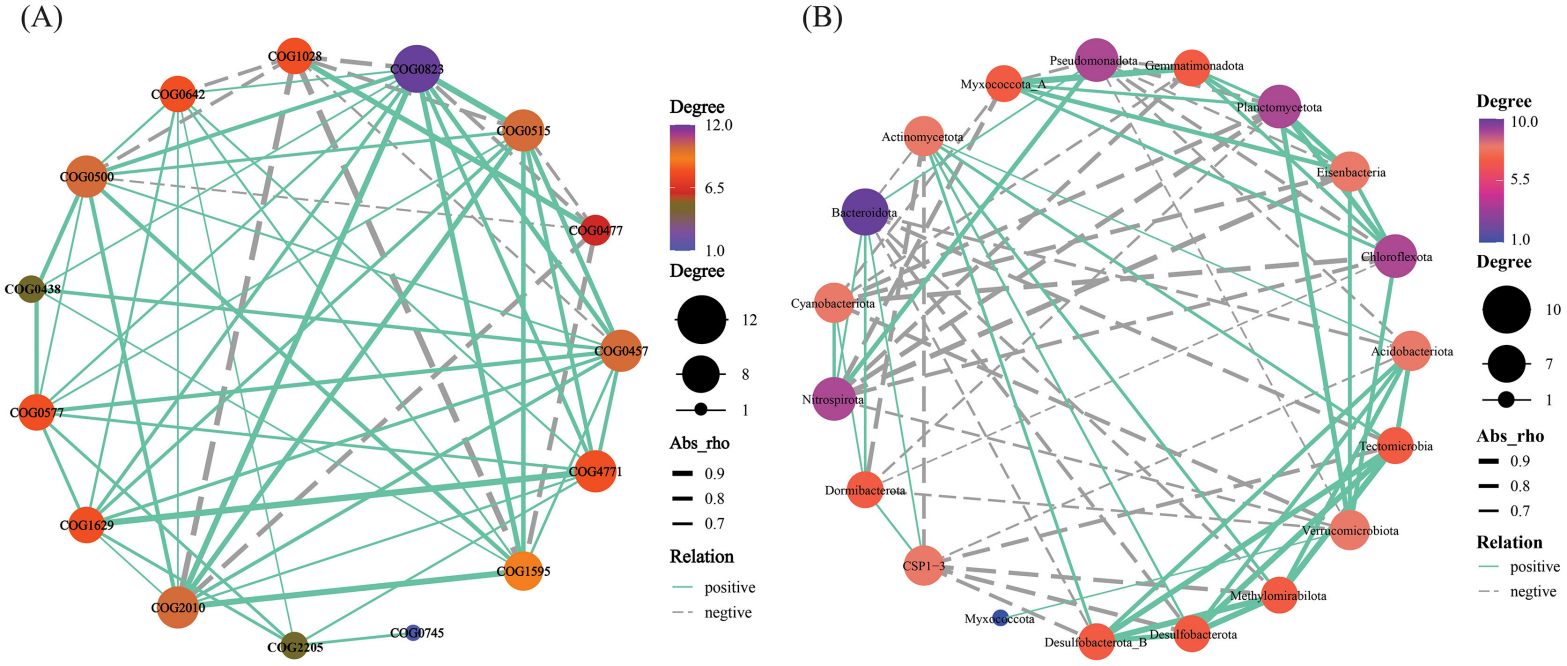
Top 15 functional genes **(A)** and top 20 soil microbial community at phylum level (**B**) correlation network diagram. This link is indicated by node connections (Spearman correlation coefficient, *r* = 0.6; *p* = 0.05). The abundance is proportional to the node size. Supplementary Table S1 displays each category's functional gene database ID, function, category, and description.

Variations in the RA of numerous functional genes in the soil microbial community are induced by CC. Sample clustering analysis of heat maps was used to group samples from the same consecutive planting years. There were significant differences between CK and other groups. The most enormous RA disparity in various functional genes was between the CK and T3 groups (Figure 5). Functional genes interact significantly with the community makeup of soil microbial communities. COG0823 was substantially more abundant than the other functional genes. It was favorably associated with many functional genes, with a stronger association with COG2010, COG1595, and COG0515. It showed a negative correlation with COG1028 and COG0477 (Figure 6A). Furthermore, COG0500, COG0515, COG0457, and COG2010 have intricate network connections and interact with several functional genes. However, the COG0745 network link is apparent and positively correlated with COG2205. Bacteroidota, the most prevalent phylum, had a negative correlation with Tectomicrobia but a positive correlation with Cyanobacteriota, Nitrospirota, and CSP1–3 (Figure 6B). Myxococcota's network interaction is quite essential and positively correlated to Verrucomicrobiota.

### Effects of soil properties and enzyme activities on microbial composition and functional genes

**Figure 7:**
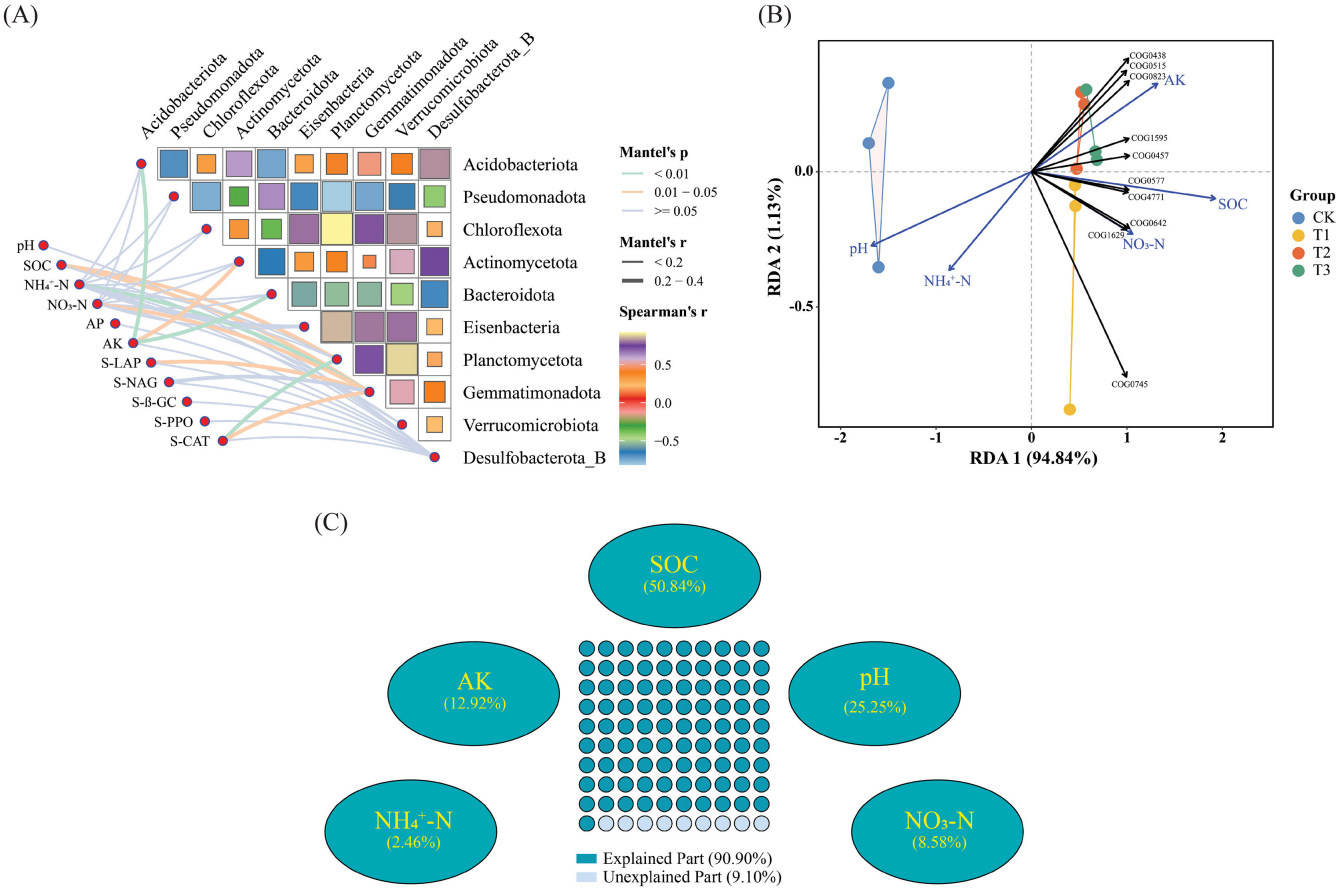
Soil chemical assays and enzyme activities influence microbial composition and functional genes. **(A)** Mantel-test analysis demonstrates the relationship between dominating microbial diversity at the phylum level and soil chemical characteristics and enzyme activities. **(B)** RDA (Redundancy analysis) analysis reveals the effects of soil chemical assays and enzyme activities on functional genes. **(C)** VPA analysis shows the contribution of soil chemistry and enzyme activities to the diversity of dominant microbial communities. Supplementary Table S1 displays each category's functional gene database ID, function, category, description, and meaning.

Changes in soil properties and enzyme activities are substantially correlated with the diversity of the dominant Phyla of microorganisms due to CC. Among them, AK is associated with Acidobacteriota and Bacteroidota, ${\text{NH}}_{4}^{+}$-N is associated with Gemmatimonadota, and CAT is associated with Planctomycetota, and their relative abundances are remarkably significantly correlated (*p* < 0.01); SOC is associated with Gemmatimonadota and Planctomycetota, NO_3_-N is associated with Gemmatimonadota, AK is associated with Actinomycetota, and LAP and Gemmatimonadota, CAT and Gemmatimonadota, whose relative abundances were significantly correlated (*p* < 0.05) (Figure 7A). RDA study revealed that soil physiochemical variables influence the number of functional genes in microbial communities. We implemented redundancy analysis to investigate the influence of environmental assays on functional genes. In order to reduce the collinearity of independent variables, we gradually eliminated variables with the most significant variance inflation factors (VIF) until the VIF of all remaining variable values was <20. Subsequently, we performed hierarchical segmentation of these factors to quantify the explanatory degree of different independent variables. Five major environmental factors (pH, SOC, ${\text{NH}}_{4}^{+}$-N, NO_3_-N, AK) affecting functional genes were screened. The first two axes accounted for 94.84% and 1.13% of the total change in the model, while the explanatory variables accounted for 99.97% (Figure 7B). AK, SOC, and NO_3_-N correlate positively with the first ranking axis, and pH and ${\text{NH}}_{4}^{+}$-N negatively correlate with the first ranking axis. pH, SOC, and AK are environmental factors with a large contribution rate, and the functional genes of each treatment are mainly affected by these three soil factors. pH is negatively correlated with the gene abundance of TOP10; SOC is highly positively correlated with the gene abundance of COG0577 genes involved in cellular processes and signal transduction and COG4771 genes involved in metabolic processes; AK is positively correlated with the abundance of multiple functional genes, the most relevant one is the COG0823 gene involved in cellular processes and signal transduction. While, VPA analysis based on Hierarchical Partitionin determined the degree of influence of various soil factor variables on microbial community diversity at the phylum level. SOC, pH, and AK had the most significant impact on microbial community diversity at the phylum level, accounting for 50.84%, 25.25%, and 12.92%, respectively (Figure 7C).

### Co-occurrence network and rhizosphere microorganisms stability

**Figure 8:**
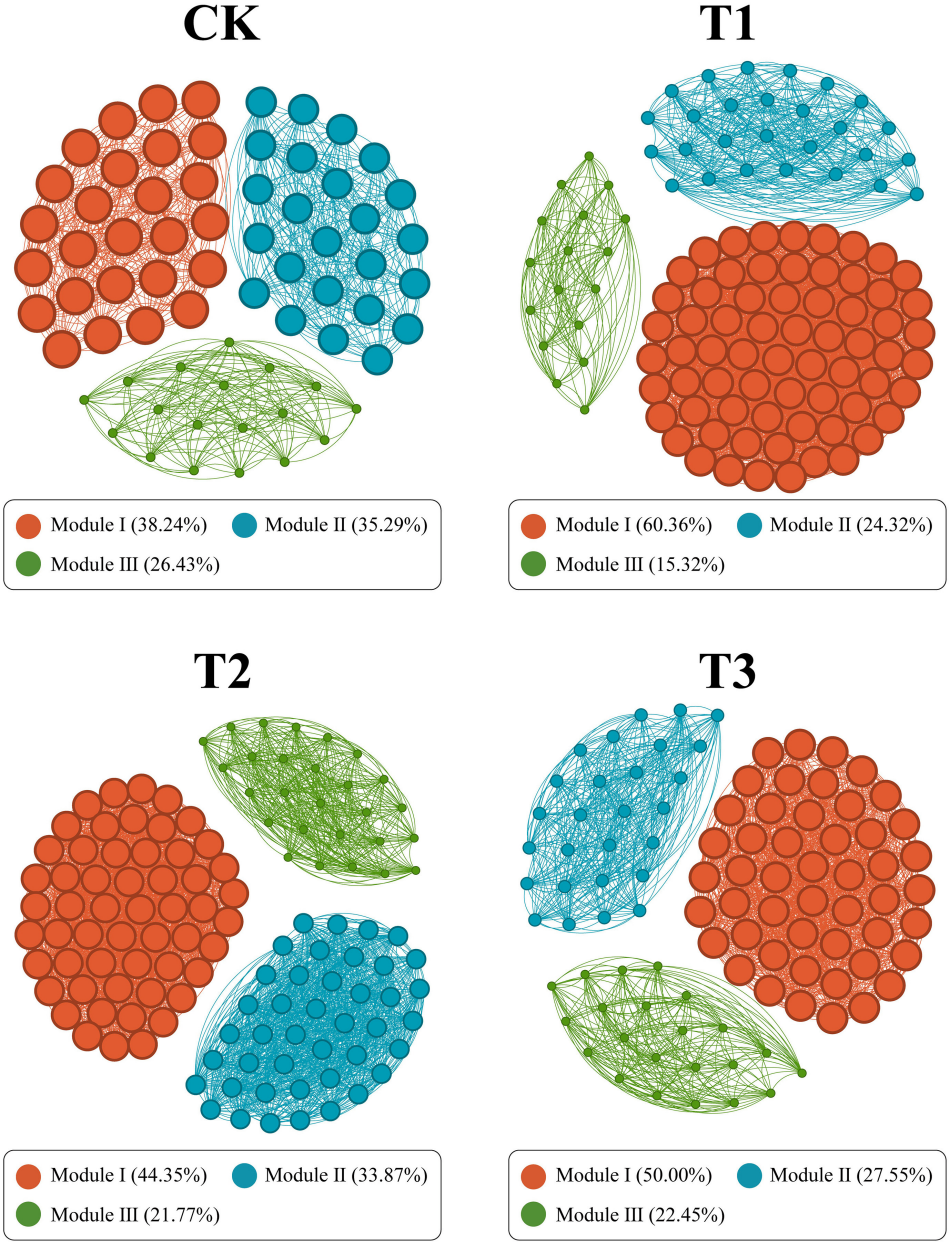
The co-occurrence network is constructed based on patchouli rhizosphere microorganisms' grade coefficient (r) at the species level under CC. Through network analysis, the co-occurrence pattern of patchouli rhizosphere microbial communities under different CC years is revealed. Nodes are colored according to different modularities. The size of each node is proportional to the degrees. A join indicates a strong (SparCC |r| > 0.5) and significant (*P* < 0.05) correlation.

**Figure 9:**
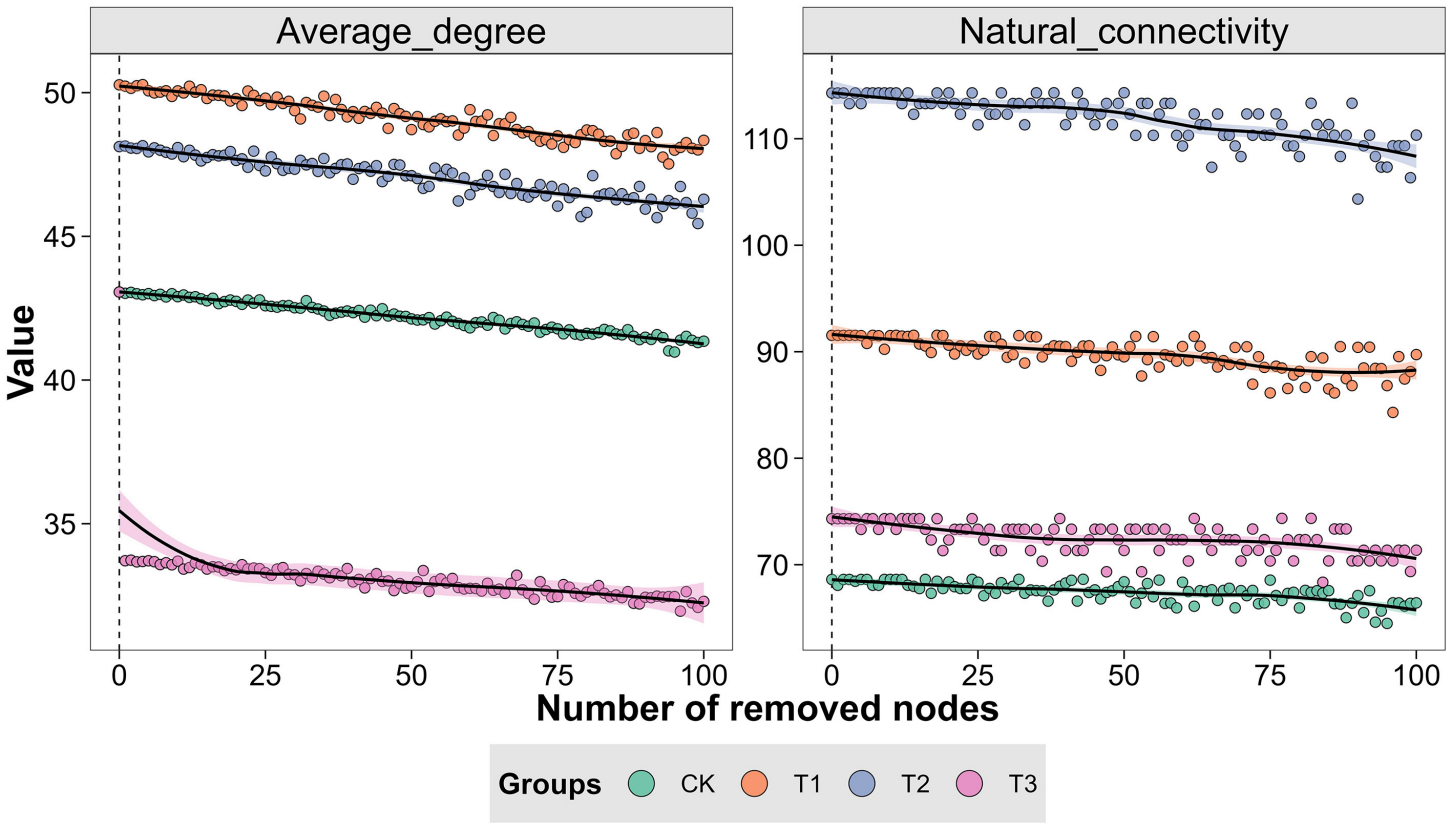
Average degree and network robustness during network invulnerability test in the CC of patchouli.

The Spearman rank correlation method generated a symbiotic network to identify the interactions between microorganisms in CC patchouli at the modular level. Every point and line in the network relates to a species of patchouli and interactions among species. The network structure's colors reflect module-level clustering products of microbial interactions. According to co-occurrence network analysis, nodes are necessary for establishing a modular structure since the probability of coexistence of microorganisms within the same module is increased. The microbial network in the patchouli rhizosphere showed different patterns in different CC years (Figure 8). This study divided the rhizosphere soil microorganisms of CC patchouli into four main modules: CK, T1, T2, and T3. The stability of the microbial communities of T1 and T2 was higher than CK and T3, respectively (Figure 9).

## Discussion

### Soil physiochemical properties and enzyme activities

CC obstacles not only affect plant growth but also retard root growth and development over the years (Zeeshan Ul Haq et al., 2023). Similarly, in patchouli plants, CC changed the soil composition and enzyme activities, which usually led to an imbalance of soil nutrients and increased pathogenic microorganisms (Zeng et al., 2020). It also stimulated the plant's roots to exude allelopathic substances, making the soil more susceptible to damage and invasion by infectious diseases (Liu et al., 2022; Zeeshan Ul Haq et al., 2023). The environmental factors in rhizosphere soil changed under the influence of CC, in which pH and ${\text{NH}}_{4}^{+}$-N decreased year after year in our results. At the same time, the concentration of AP increased with the CC years, but NO_3_-N content observed fluctuations with the passing years (Table 1). This decrease in soil pH and ${\text{NH}}_{4}^{+}$-N can negatively impact nutrient availability, alter microbial communities, and potentially increase metal toxicity, nitrogen deficiency in plants, disrupt the overall nitrogen cycle, and influence soil microbial composition in the soil (Barth et al., 2019; Gu et al., 2022; Wang F. et al., 2024; Wang K. et al., 2024). NO_3_-N and AP fluctuations can lead to inconsistent nitrogen availability for plants, affecting growth and yield stability across cropping years (Ferretti et al., 2021). The enzyme activities of the rhizosphere soil are also affected by CC. Among them, the four enzyme activities of LAP, NAG, CAT, and GC showed a significant increase compared with CK at the first 2 cropping years (T1 and T2), then decreased at the third (T3), while PPO increased year after year in CC. The CC might alter soil microbial community diversity and composition, raising the changes in pathways (microbial metabolic) and altering the soil enzyme activities (Liu et al., 2022). This suggested that while initial years of cropping may stimulate specific soil biological processes, prolonged CC can lead to alterations in soil biochemical functions (Qu et al., 2022).

### Soil microbial abundance and composition in continuous cropping

The total microbial communities in patchouli rhizosphere soil in CC accounted for 91.38%, of which the bacterial community was dominant (RAavg, 88.8%), and the RA difference between the four treatments was minimal. The RA of eukaryotes and viruses decreased significantly in CC (Figure 1). This suggests that eukaryotes and viruses are more susceptible to variations in the soil environment than bacteria in the soil. A study on the change of environmental factors on eukaryotic communities in soil found that eukaryotes are more sensitive to temperature and humidity alterations than bacteria in the soil (Zhao et al., 2018). Similarly, another investigation discovered that land use patterns significantly influence the structure and functionality of the soil virus communities (Liao et al., 2022). These findings suggest that eukaryotes and viruses were more susceptible to changes in the soil environment than bacteria. Environmental factors play a crucial role in shaping their communities and functions. After CC, the patchouli rhizosphere microbial community has undergone significant changes. From the perspective of dominant microorganisms at the phylum level, compared with CK, the RA of Acidobacteriota, Chloroflexota, Actinomycetota, and Eisenbacteria in CC soil increased, and Pseudomonadota and the RA of Bacteroidota decreased (Figure 2). The increase in these phyla suggests an adaptation of the soil microbial community to the specific conditions, including Acidobacteriota might induced by acidic conditions (Zeng et al., 2020), Chloroflexota increased might be due to the accumulation of specific organic substrates from patchouli root exudates (Wang F. et al., 2024; Wang K. et al., 2024), Actinomycetota induced due to decomposition activities in the rhizosphere (Yao et al., 2020), and Eisenbacteria associated with the organic matter decomposition which aligns with the continuous input of plant-derived materials in monoculture systems (Li et al., 2024) created by CC of patchouli. Similarly, RA of Pseudomonadota is often associated with plant growth promotion and nutrient cycling (Yao et al., 2020; Zeng et al., 2020). Bacteroidota is known for degrading complex organic compounds, and its reduction could affect soil organic matter dynamics (Zeng et al., 2020; Sui et al., 2023). These changes are consistent with observations in other CC systems, such as the similar shifts in bacterial communities reported by Liu et al. in continuously cropped cucumber rhizosphere soil (Yao et al., 2020). There are 1898, 127, 42, and 44 unique microbial genera in CK, T1, T2, and T3, respectively (Figure 4). At the genus level, the number of unique microbial genera in the soil significantly decreased across CC years. Studies have shown that as the duration of CC increased, the number of unique microbial genera decreased prominently, leading to a loss of overall microbial diversity and soil functional capacity (Huang et al., 2023). This observation is consistent with other research, indicating that CC reduces microbial diversity in *Cucumis sativus* and *A. hypogaea* (Zhou et al., 2020; Yao et al., 2020).

### Soil microbial functions in continuous cropping

After CC, the soil's RA of various functional genes changed significantly. Among these changes, the difference in RA between the CK and T3 was the most pronounced. The data revealed a significant interaction between functional genes and the composition of soil microbial communities (Figure 5). This interaction is crucial as it implies that changes in the abundance of certain functional genes can influence the overall structure and function of the microbial community. For instance, research has demonstrated that the diversity and abundance of genes substantially affect the structure and function of microbial communities (Jiang et al., 2019). This dynamic relationship highlighted the importance of understanding how different functional genes interact within the soil composition (Cong et al., 2015; Waldrop et al., 2023). The functional gene COG0823, associated with cell signal transduction (Supplementary Table S1), exhibits significantly higher abundance than other functional genes (Figure 6A), highlighting its core role in soil microbial networks. Previous research suggested this gene is involved in bacterial pathogenicity (Szczepaniak et al., 2020). Furthermore, COG0823 is crucial in nutrient cycling, decomposition, and other essential microbial processes (Ge et al., 2018). Studies showed that soils with a high abundance of COG0823 host-specific microbial groups associated with nutrient cycling exhibit increased populations. This suggests a synergistic relationship that enhances soil fertility (Ding et al., 2024). In contrast, COG0745 demonstrated a relatively simple network relationship, being positively correlated only with COG2205 (Figure 6A). The limited interactions of COG0745 suggest it may play a specialized role within microbial communities (Ma et al., 2022). The positive correlation between COG0745 and COG2205 indicates a potential cooperative relationship between these two genes, which could be critical for specific ecological functions, such as facilitating symbiotic interactions or supporting shared metabolic pathways (Wu et al., 2005, 2020). For instance, the metabolic pathways involving COG0745 may rely on the material transport facilitated by COG2205. This positive correlation underscores their potential functional connections and highlights their significant roles in soil ecosystems. Future research could further investigate the specific molecular mechanisms underlying this relationship and its implications for soil health and ecological balance.

### Correlation analysis of dominant microbial communities

In continuous patchouli cropping systems, microbial communities exhibit notable interactions and correlations at the phylum level. Specifically, Bacteroidota, which had the highest abundance, was negatively associated with Tectomicrobia and positively associated with Cyanobacteriota, Nitrospirota, and CSP1-3. Additionally, the network relationship of Myxococcota was comparatively easy, being only positively associated with Verrucomicrobiota (Figure 6B). Bacteroidota, a dominant phylum in the patchouli rhizosphere, is crucial in soil health and nutrient cycling. The negative correlation with Tectomicrobia suggests competitive or antagonistic interactions, possibly due to competition for similar ecological niches or resources. Conversely, the positive correlations with Cyanobacteriota, Nitrospirota, and CSP1-3 indicate potential synergistic relationships. Cyanobacteria are known for their nitrogen-fixing capabilities, which could benefit Bacteroidota by enhancing soil fertility. Similarly, Nitrospirota, involved in nitrogen cycling, might create a favorable environment for Bacteroidota through their metabolic activities (Zeng et al., 2020; Yang et al., 2022; Kamada et al., 2023). The Myxococcota positive association with Verrucomicrobiota could be indicative of a mutualistic relationship where both phyla benefit from each other's presence, possibly through nutrient exchange or habitat modification (Kamada et al., 2023; Li J. et al., 2023; Li L. et al., 2023; Li Z. et al., 2023). The CC altered soil physiochemical properties and enzyme activities, significantly impacting the diversity and relative abundances of dominant microbial phyla, including AK and Acidobacteriota and Bacteroidota, ${\text{NH}}_{4}^{+}$-N and Gemmatimonadota, and CAT and Planctomycetota, with a significant correlation (*p* < 0.01) (Figure 7A), verified by the previous studies (Alami et al., 2021; Zhang et al., 2020; Yang et al., 2023; Song et al., 2024; Wang F. et al., 2024; Wang K. et al., 2024). RDA analysis between functional genes and environmental factors revealed that pH, SOC, and AK are key factors influencing the function of microbial communities (Figure 7B). Research has demonstrated that soil pH influences the bacterial communities in agricultural soils more significantly than nutrient content. Changes in pH can alter the microbial mechanisms of carbon accumulation and decomposition, impacting SOC dynamics (Malik et al., 2018; Wang et al., 2019; Kang et al., 2021). The microbial network in the patchouli rhizosphere exhibited different patterns across various CC years (Figure 8). CC decreased the stability of the patchouli rhizosphere microbial community, with the stability of T1 and T2 being higher than that of T3 and CK (Figure 9). The reduced stability of long-term CC suggests that extended CC may hinder key soil functions (disease suppression, nutrient cycling, and decomposition of organic matter) to lower soil fertility and crop production (Liu et al., 2020; Fox et al., 2022; Li J. et al., 2023; Li L. et al., 2023; Li Z. et al., 2023). Research showed that in CC systems, the reduction of microbial diversity leads to a weakened ability of soil to suppress diseases, consequently increasing the incidence of crop diseases (Olanrewaju and Babalola, 2022; Cao et al., 2024). The CC shortens microbial community structures, reduces the efficiency of organic matter decomposition, and ultimately results in decreased soil fertility (Yuan et al., 2021). The potential mechanisms underlying these phenomena include decreased nutrient availability. The CC can deplete essential nutrients in the soil or alter their availability, which may reduce the populations of beneficial microorganisms responsible for disease suppression and nutrient cycling. Studies have reported a correlation between improved soil nutrient status and enhanced disease suppression capabilities (Cao et al., 2024). In stable ecosystems with high microbial diversity, beneficial microorganisms effectively compete with pathogens for resources. However, pathogens may proliferate unchecked in systems where unique microbial genera are lost due to CC (Zhou et al., 2020). This shift increases disease pressure on crops and disrupts nutrient cycling processes mediated by beneficial microbes. Based on this study, we suggest that future efforts can focus on developing bio-fertilizers and monitoring and controlling environmental factors that significantly increase or decrease after CC that can alleviate CC obstacles.

## Conclusions

In summary, CC is an unavoidable farming method in patchouli cultivation and production. On the other hand, prolonged CC alters the rhizosphere microorganisms while negatively affecting the physiochemical properties of the soil, enzyme activities, and microbial communities structure and their functions; after that, the microbial community is reduced. The microbial community structure of the patchouli rhizosphere soil was significantly altered by CC, as bacteria and archaea are more susceptible to adapting to changes in soil conditions than eukaryotes and viruses. The primary environmental factors influencing the makeup of the abundance of microbial community function genes are soil pH, SOC, and AK. Each successive year of CC diminishes microbial community stability in the patchouli rhizosphere. These findings showed that CC alters the structure and functions of the microbial communities and the soil's physiochemical and enzyme activities. Additionally, as CC increases, the stability of the microbial community declines. This work established a theoretical foundation for the obstacles faced in continuously cultivating patchouli. It comprehensively assessed the impacts of CC on the soil assays and microbial community structure in the patchouli rhizosphere.

## Data Availability

The datasets presented in this study can be found in online repositories. The names of the repository/repositories and accession number(s) can be found below: https://www.ncbi.nlm.nih.gov/, PRJNA1191635.
