# Supplementary figures and images for: Continuous cropping of Patchouli alters soil physiochemical properties and rhizosphere microecology revealed by metagenomic sequencing
Source: Front Microbiol. 2025 Jan 13;15:1482904. doi: 10.3389/fmicb.2024.1482904 (PMC11769982; doi:10.3389/fmicb.2024.1482904)

**Supplementary Figures**


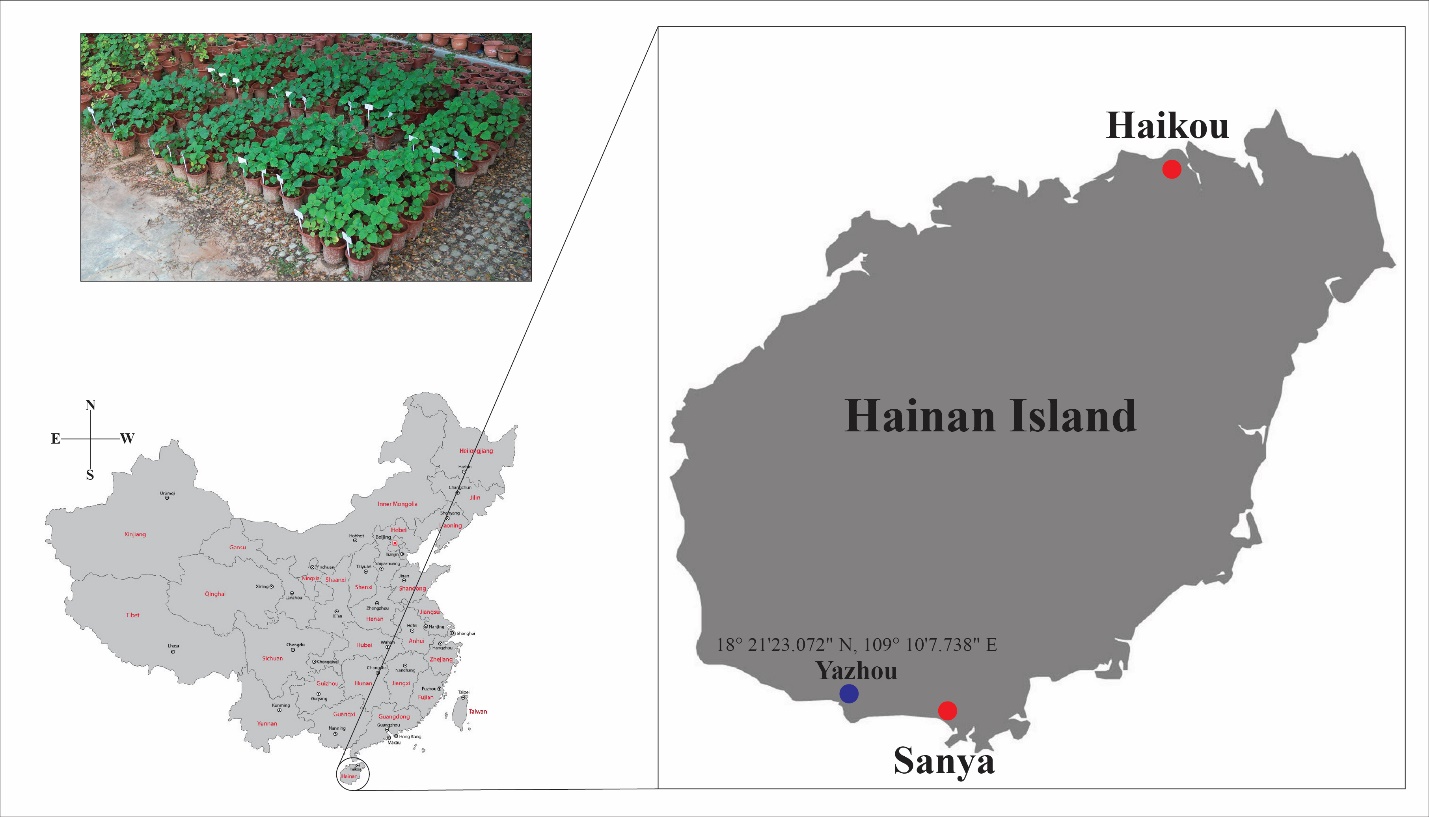


**Figure S1.** Experimental layout and its location.

Supplement: Supplementary file 2 [file Data_Sheet_1.docx]
